# Substances in the Nose

**Published:** 1875-01

**Authors:** 


### SUBSTANCES IN THE NOSE.

A kernel of corn, or a pea, or other small substance, gets into a child's nose, and the problem is to remove it. A recent medical journal proposes to administer an emetic ; and then, at the moment vomiting is about to begin, press a handkerchief, or napkin, firmly over the mouth. The vomited matter must then pass through the nose, and the force of its current will wash away the intruding substance. We have not seen this plan tried, and cannot vouch for its efficacy or safety. It seems to us that the volume of the discharge from the stomach would be too great to pass through the nose, even if both nostrils were unobstructed. If so, regurgitation into the œsophagus and larynx with consequent strangling would result.

We would suggest the following method as less objectionable and quite as effectual in accomplishing the object desired. Inject into the free nostril with a common syringe, a stream of tepid water. The sides of the nostril should be closed around the point of the syringe to prevent a re-flux of the water. Use a moderate degree of force and the stream will make a circuit through the posterior nasal cavity and pass out of the opposite nostril dislodging the foreign substance.

A more simple method is this. The patient takes a "deep breath," then closes the mouth and the free nostril and forces his breath through the obstructed nostril. If the child is old enough to perform this little feat perfectly he may blow out the corn, or pea, or whatever has found a lodgment in the nasal passage.

---

Calomel.—As a proof of the effect of medicine persistently taken, it is stated that Professor Hyatt, of Vienna, threw down a bone on a table before his students with such violence, that it broke into several pieces, when it was noticed that little globules of mercury were dancing about over the table. The man of whom this bone was once a part, had taken a great deal of mercury during the year previous to his death.

---

To Renovate Furniture. — The handiest thing which can be kept about a house as a general renovator, is a solution of gum-shellac in alcohol. It costs but a trifle, and a pint will last for years. Put the two in a bottle and in a couple of days you have a brown, creamy varnish, which you can apply with a small brush. It dries in two minutes, and leaves a most brilliant surface. If you want the French polish you must add an equal amount of sweet oil, and shake well each time you use it. You do not use a brush for this mixture, but put it on a bit of cotton flannel and rub over quickly in only one direction, being careful not to continue the rubbing long enough to take the shine off. Sometimes the humidity of the atmosphere and the action of the gas cause a bluish white coating to collect on all furniture, which shows conspicuously on bright, polished surfaces, such as mirrors, pianos, cabinet ware and polished metal. To remove it, take a soft sponge, wet with clean cold water, and wash over the article. Then take a soft chamois skin and wipe it clean. Dry the skin as well as you can by wringing it in the hands and wipe the water off the furniture, being careful to wipe only one way. Never use a dry chamois on varnish work. If the varnish is defaced and shows white marks, take linseed oil and turpentine in equal parts ; shake them well in a phial and apply a very small quantity on a soft rag until the color is restored; then with a clean, soft rag wipe the mixture off. In deeply carved work, the dust cannot be removed with a sponge. Use a stiff-haired paint brush instead of a sponge.

To varnish old furniture, it should be rubbed with pulverized pumice-stone and water to take off the old surface, and then varnished with varnish reduced by adding turpentine, to the consistency of cream. Apply with a stiff-haired brush. If it does not look well, repeat the rubbing with pumice-stone, and when dry, varnish it again.

